# Efficacy and safety of Danhong injection for treating myocardial infarction: a systematic review and meta-analysis of randomized controlled trials

**DOI:** 10.3389/fphar.2024.1371959

**Published:** 2024-06-13

**Authors:** Shiyi Yang, Yin Wang, Hailiang Shen, Tianhang Chen, Haixia Du

**Affiliations:** ^1^ College of Life Science, Zhejiang Chinese Medical University, Hangzhou, China; ^2^ College of Basic Medical Science, Zhejiang Chinese Medical University, Hangzhou, China

**Keywords:** Danhong injection, myocardial infarction, traditional Chinese medicine, cardiovascular disease, meta-analysis

## Abstract

**Objective:**

Danhong injection (DHI) is widely used in the treatment of myocardial infarction (MI). We aimed to systematically review the efficacy and safety of DHI in a randomized controlled experiment on MI.

**Methods:**

We searched the randomized controlled trials (RCTs) of DHI for MI published before 2 April 2023 in China National Knowledge Infrastructure (CNKI), Chinese Biomedical Literature Database (CBM), Wanfang database, China Science and Technology Journal Database (VIP), PubMed, Web of Science, Cochrance Library, and Embase databases. The methodological quality of the included studies was evaluated using the Cochrane Handbook 5.3 criteria using the RevMan software, and meta-analysis was performed and a forest map was drawn.

**Results:**

A total of 38 trials included 3877 patients, including 2022 cases in the DHI treatment group and 1855 cases in the control group. Meta-analysis showed that the total effective rate (RR = 1.18%, 95% CI [1.14–1.12]) during treatment with DHI was higher than that of the control group. The prevalence of cardiac arrhythmia (RR = 0.55%, 95% CI [0.46–0.65]) was lower than that of the control group. The incidence of heart rate failure (RR = 0.45%, 95% CI [0.30–0.70]) was lower than that of the control group. The prevalence of cardiogenic shock (RR = 0.33%, 95% CI [0.11–1.04]) was *p* > 0.05, and the difference was not statistically significant. There was no statistically significant difference in LVEF between the two groups (MD = 0.00%, 95% CI [0.00–0.00]). CK-MB (MD = −0.81%, 95% CI [−0.92∼ −0.69]) was lower than the control group. hs-CRP (MD = −1.09, 95% CI [−1.22∼ −0.97]) was lower than the control group. The incidence of adverse reactions (RR = 0.37, The 95% CI [0.17–0.82]) was lower than that in the control group.

**Conclusion:**

Basing on our study, the use of DHI in the treatment of myocardial infarction patients is effective, can improve cardiac function, reduce the incidence of adverse reactions, and improve the overall quality of life.

**Systematic Review Registration:**

https://www.crd.york.ac.uk/PROSPERO/, identifier CRD42023390973.

## Introduction

Myocardial infarction (MI) refers to myocardial ischemia and necrosis, which is a kind of acute and critical illness based on coronary artery disease (Hu et al., 2012). After MI, many serious complications may occur, including muscle rupture, septal compartment, and free wall rupture (Murphy and Goldberg, 2022), as well as malignant arrhythmias such as cardiogenic shock, heart failure, or premature ventricular contractions, etc. MI has a high incidence of acute attacks and a high mortality rate, making it a common cause of death from coronary heart disease (Zhang et al., 2019). In recent years, the age of onset has tended to be younger (Song et al., 2023). According to data released by the National Health Commission, cardiovascular disease (CVD) ranks first in the mortality rate of diseases in China (Yang et al., 2023).

The general treatment methods for MI include coronary intervention (PCI), emergency thrombolytic therapy, elective surgery and drug therapy. However, these treatment methods still have some shortcomings, such as most hospitals using selective PCI in the timing of PCI intervention, but there is a conflict with the treatment principles of ST-elevation myocardial infarction (STEMI) in the timing of intervention (Hu et al., 2023). Due to the increased risk of bleeding, emergency thrombolytic therapy is usually contraindicated in certain conditions. In addition, thrombolysis seems to have little effect on the prognosis of patients with cardiogenic shock or venous graft occlusion (Himbert et al., 1995). At present, the intervention and drug treatment of MI in China have not yet reached a certain level, and the high cost is also a problem. Therefore, there is an urgent need to explore other potentially effective interventions to treat MI.

Traditional Chinese medicine (TCM) believes that the main pathogenesis of MI is heartache, true heartache, and chest paralysis. The treatment method mainly focuses on supplementing qi and promoting blood circulation, and TCM treatment can not only significantly improve clinical symptoms, but also improve objective parameters and increase clinical cure rate (Gao et al., 2018). Danhong injection (DHI), as one of the most popular clinical TCM medications for the treatment of MI, is extracted from Danshen (*Salvia miltiorrhiza* Bunge) and Safflower (*Carthamus tinctorius* L.), which are prepared in a ratio of 3:1 (Wang et al., 2022), It has good effects on promoting blood circulation, removing blood stasis, and relieving pain through menstruation. DHI has anti-atherosclerosis, inhibiting platelet aggregation (Li et al., 2022), improving microcirculation, promoting fibrinolysis, and even has a certain anticoagulant effects (Feng et al., 2019). Research has shown that DHI is superior to Western medicine in terms of incidence of adverse cardiac reactions, promotion of reperfusion, improvement of heart function, and protection of myocardium (He et al., 2021). However, there is limited literature review and systematic review on the treatment of MI with DHI, which requires further research.

In this study, we conducted a systematic and comprehensive analysis of relevant randomized controlled trials (RCTs) of DHI treatment for MI through meta-analysis, so as to better verify the efficacy and safety of DHI treatment for MI.

## Methods

This study was conducted following the protocol registered with the International Prospective Register of Systematic Reviews (PROSPERO) under the number CRD42023390973. Our meta-analysis was performed in accordance with the Preferred Reporting Item for Systematic Reviews and Meta-analyses (PRISMA) guidelines (Bernardo, 2017).

### Eligibility and exclusion criteria

The randomized controlled trials (RCTs) consistent with the following requirements were taken into our consideration: 1) Languages are limited to Chinese and English. 2) The subjects of the study were MI patients with no age or gender restrictions. 3) In the experimental group, Danhong injection was added to the basic treatment of the control group with 5% glucose solution or 0.9% sodium chloride injection as an intravenous infusion (Dezawa et al., 2019). 4) The control group was treated with conventional treatment, including oxygen inhalation, analgesics, thrombolysis, nitrates, lipid-regulating drug atorvastatin (Hu et al., 2021), β-receptor blocker (Pitcher et al., 2022), antiplatelet aggregation drug ticagrelor (Wang et al., 2018), anticoagulant drug low molecular weight heparin, unfractionated heparin, etc. Research design: randomized controlled trial.

Studies were excluded according to the following criteria: 1) Duplicate publications, and only one of them remains, 2) Reviews, systematic reviews, reviews, animal tests, etc., 3) It is inconsistent with the research content of this paper, 4) The experimental methods do not match, 5) The type of study was non-RCT, cohort study or case-control study, 6) outcome measures were inconsistent, 7) those obvious flaws, such as data duplication or statistical errors, and 8) There is only a summary and no complete article.

### Literature sources and search

Computer search databases: China National Knowledge Infrastructure (CNKI), Chinese Biomedical Literature Database (CBM), Wanfang database, China Science and Technology Journal Database (VIP), PubMed, Web of Science, Cochrance Library, and Embase databases. The included studies were published between the establishment of each database and 2 April 2023. Search terms include “Danhong,” “Danhong Injection” and “Myocardial Infarction”.

### Evaluating indicator

The primary outcome was the total effective rate. The secondary outcomes were the incidence of arrhythmia (Krahn et al., 2022), the incidence of heart rhythm failure, and the incidence of cardiogenic shock; Left ventricular ejection fraction (LVEF) (Bozkurt et al., 2021); Creatine kinase isoenzyme (CK-MB) (Minamidate et al., 2022); Hypersensitive C-reactive protein (hs-CRP) (Kandelouei et al., 2022); Incidence of adverse reactions (ADRs).

### Literature inclusion and data Extraction

The resulting literature library was created using EndNote 9.1 software, and the inclusion of the literature was independently completed by two researchers. If the results were disputed at any stage, a third researcher would be involved in the discussion. In addition, useful data described in detail in all the original studies included in the analysis were extracted, including basic information (author’s name, date of publication, title), detailed characteristics of included patients (sample size, sex, mean age), diagnosis standards, treatment time, intervention measures (drug), outcomes (primary outcome, secondary outcome), and information on the quality of RCTs (Jadad score).

### Risk of bias assessment

Quality assessment was carried out using the Cochrane assessment of risk of bias tool (Higgins et al., 2011), and the quality of all included RCTs was independently assessed by two review authors. Low-risk, high-risk, and unclear risk were assessed based on seven domains: randomization method, concealment of allocation method, blinding of participants and personnel, blinding of outcome assessment, completeness of outcome data, selective reporting of study results, and other sources of bias (Whiting et al., 2011; Drucker et al., 2016).

### Statistical analysis

Review Manager 5.3 software was used to create a schematic diagram of the risk assessment of bias and a schematic diagram of the proportion of each item of quality assessment. Forest plots were plotted using RevMan 5.3, and for dichotomous variables, risk ratio (RR) (Talebi et al., 2019) and 95% confidence intervals (95% CI) were used to represent the results of a meta-analysis of total effective rate, incidence of arrhythmias, incidence of heart rate failure, and incidence of cardiogenic shock. For continuous data, the mean difference (MD) and 95% confidence interval (95% CI) were used to represent the meta-analysis results for LVEF, CK-MB, and hs-CRP. Heterogeneity was included in these studies using the X^2^ test and the value of I^2^ analysis (Lin et al., 2017). When the 95% CI for RR did not include 1 or the 95% CI for MD did not contain 0, there was a significant difference in the results.

## Results

### Literature search and basic information

**FIGURE 1:**
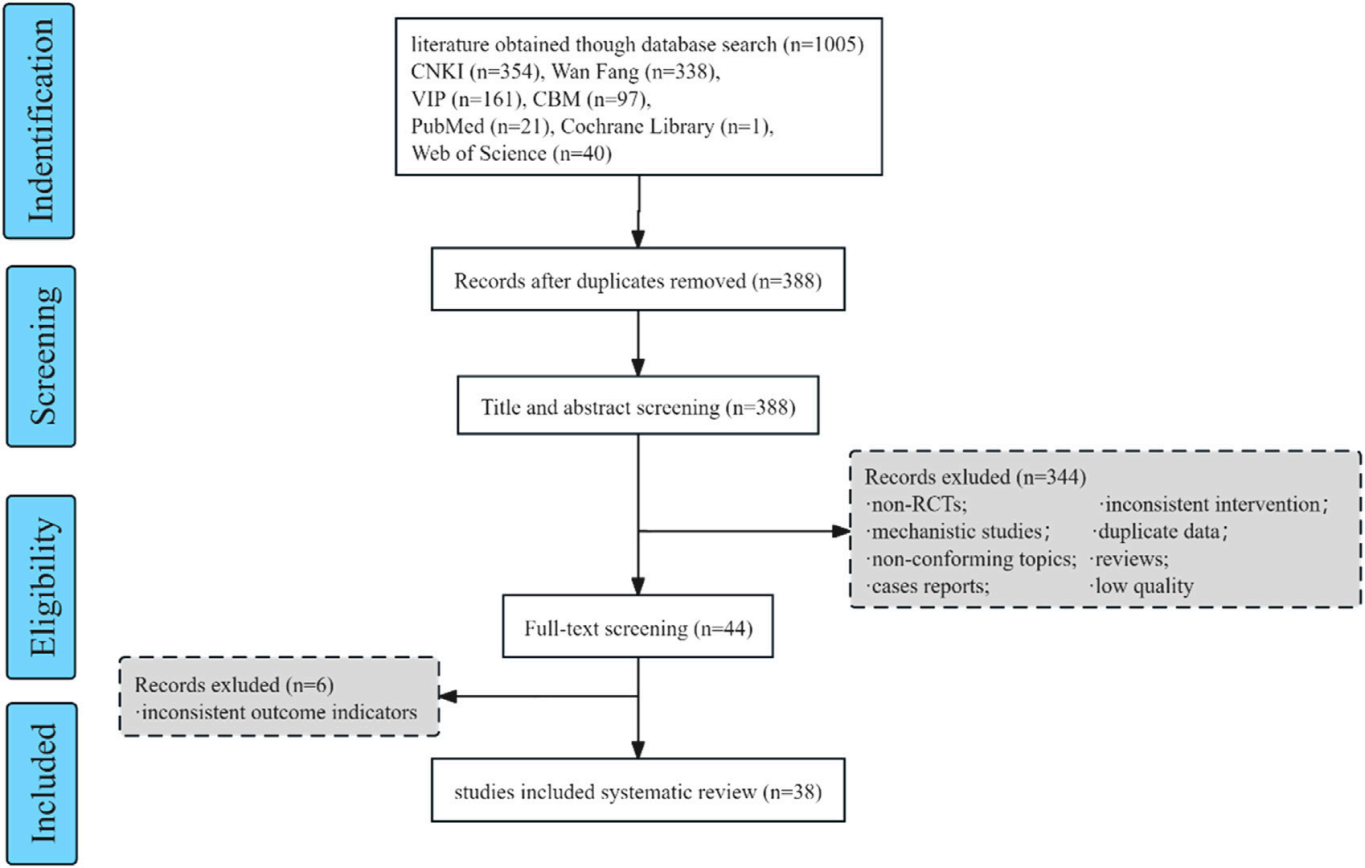
Flow chart of literature screening.

A total of 1005 articles were searched through these databases, and EndNote 9.1 software was used to screen a total of 38 articles in the meta-analysis study, and the screening details are shown in Figure 1. The 38 studies included 3877 patients, including 2022 patients in the Danhong treatment group and 1855 patients in the control group, and the basic information is shown in Table 1.

**TABLE 1:** Basic information of the included studies.

ID	Method	Object of observation			Diagnosis standards	Intervention	Treatment time	Outcomes	Jadad score
		Total sample (T/C)	Sex [T (M/F):C (M/F)]	Age (T:C)					
Chen and Liu (2013)	RCT, Open-blinding	75 (39/36)	(21/18): (19/17)	(52.3 ± 3.6): (53.1 ± 2.9)	UNMIDT (2007 CMA)	DHI (30 mL ivgtt qd)	14d	TER, ADRs	4
Li et al. (2019)	RCT, Open-blinding	90 (45/45)	(20/25): (23/22)	(60.17 ± 2.85): (60.14 ± 4.81)	NR	DHI (30 mL ivgtt qd)	14d	LVEF, ADRs	4
Geng and Hu (2016)	RCT	63 (32/31)	(23/9): (22/9)	(62.7 ± 9.2): (61.5 ± 8.9)	NR	DHI (20 mL ivgtt qd)	7d	IA, hs-CRP	5
Kong and Zhao (2014)	RCT	60 (30/30)	(16/14): (17/13)	67: 66	NR	DHI (30 mL ivgtt qd)	10d	ADRs	5
Ren and Jia (2013)	RCT	40 (20/20)	(27/13) (no details)	NR	CM (2003)	DHI (40 mL ivgtt qd)	14d	IHF, ICS, hs-CRP	5
Chen et al. (2010)	RCT, Open-blinding	59 (29/30)	(22/7): (21/9)	(62 ± 5): (56 ± 12)	GDMAMI (2001 CMA)	DHI (20 mL ivgtt qd)	14d	LVEF	5
Zeng et al. (2017)	RCT, Open-blinding	120 (60/60)	(38/22): (36/24)	(65.13 ± 2.38): (64.38 ± 2.12)	GDMAMI (2001 CMA)	DHI (40 mL ivgtt qd)	14d	TER, IHF, LVEF	5
Liu and Gu (2014)	RCT	117 (58/59)	(62/55) (no details)	NR	DTSTEMI (2010)	DHI (30 mL ivgtt qd)	14d	LVEF	5
Jin et al. (2019)	RCT	117 (60/57)	(39/21): (38/19)	(57.7 ± 5.49): (57.4 ± 6.37)	NR	DHI (40 mL ivgtt qd)	14d	LVEF	6
Wang et al. (2015)	RCT	60 (30/30)	(35/25) (no details)	NR	GDMAMI (2001 CMA)	DHI (30 mL ivgtt qd)	7d	IA, CK-MB, hs-CRP	5
Jin et al. (2011)	RCT	60 (30/30)	(16/14): (17/13)	(59 ± 7): (60 ± 10)	NR	DHI (30 mL ivgtt qd)	14d	IA, LVEF, CK-MB, hs-CRP	5
Hu et al. (2011)	RCT	64 (32/32)	(20/12): (23/9)	(50.72 ± 8.63): (50.28 ± 5.66)	NR	DHI (20 mL ivgtt qd)	7d	RBV, CFR, IA, IHF, CK-MB	5
Han et al. (2012)	RCT	134 (76/58)	(42/34): (32/26)	(55.6 ± 12.5): (51.8 ± 13.6)	GDMAMI (2001 CMA)	DHI (20 mL ivgtt qd)	14d	IA, IHF, ICS	5
Xu et al. (2015)	RCT, Open-blinding	71 (36/35)	(49/22) (no details)	(65 ± 13): (63 ± 11)	DTSTEMI (2010)	DHI (40 mL ivgtt qd)	14d	LVEF, CK-MB	5
Wu (2019)	Non-RCT, Open-blinding	120 (60/60)	NR	(62 ± 2.5): (65 ± 3.5)	NR	DHI (30 mL ivgtt qd)	7d	TER, hs-CRP	3
Cui and Wang (2014)	RCT, Open-blinding	180 (90/90)	(52/38): (54/36)	(72.1 ± 6.5): (72.3 ± 5.8)	GDMAMI (2001 CMA)	DHI (30 mL ivgtt qd)	10d	TER, hs-CRP	4
Qin et al. (2014)	RCT	112 (56/56)	(31/25): (30/26)	(52.31 ± 11.24): (55.12 ± 10.52)	DCAMI (1979 WHO)	DHI (40 mL ivgtt qd)	7d	CHF, CS, LVEF, CK-MB, hs-CRP	5
Zhang et al. (2019b)	RCT, Open-blinding	80 (40/40)	(46/34) (no details)	(69.88 ± 3.16): (68.92 ± 3.08)	DTSTEMI (2015)	DHI (30 mL ivgtt qd)	7d	CFR, IA	4
Wang et al. (2009)	RCT	203 (116/87)	(66/50): (45/42)	(71.6 ± 8.6): (70.7 ± 8.1)	NR	DHI (30 mL ivgtt qd)	14d	TER	5
Li et al. (2022a)	RCT, Open-blinding	82 (41/41)	(25/16): (24/17)	(62.5 ± 4.6): (62.3 ± 4.5)	NR	DHI (20 mL ivgtt qd)	7d	TER, BPM, CHF, LVEF, hs-CRP	5
Yan (2013)	RCT	160 (80/80)	(52/28): (55/25)	(61.5 ± 18.4): (59.5 ± 19.5)	CM (1980 NCPGIM)	DHI (30 mL ivgtt qd)	14d	TER	5
Jia et al. (2013)	RCT	65 (33/32)	(21/12): (19/13)	(61.28 ± 13.76): (62.74 ± 11.19)	CHD (2003)	DHI (30 mL ivgtt qd)	14d	TER, hs-CRP	5
Zhang et al. (2018)	RCT, Open-blinding	110 (55/55)	(33/22): (35/20)	(61.9 ± 4.9): (62.5 ± 4.2)	DCASTEMI	DHI (20 mL ivgtt qd)	14d	TER, CK-MB	5
Ji et al. (2016)	RCT, Open-blinding	108 (54/54)	(38/16): (36/18)	(62.3 ± 6.5): (61.8 ± 6.7)	GTNSTE-ACS (2012)	DHI (40 mL ivgtt qd)	14d	TER, IA, ICS, CK-MB	4
Zhou et al. (2010)	RCT	65 (33/32)	(18/15): (17/15)	(64.5 ± 6.3): (62.5 ± 8.4)	DCAMI (1979 WHO)	DHI (30 mL ivgtt qd)	14d	LVEF	5
Bai et al. (2009)	RCT	80 (45/35)	(30/15): (17/18)	NR	NR	DHI (20 mL ivgtt qd)	10d	TER	5
Qi et al. (2008)	RCT	40 (20/20)	(12/8): (13/7)	(63.9): (68.1)	NDCIHD (WHO)	DHI (20 mL ivgtt qd)	20d	TER	5
Han and Wang (2012)	RCT	64 (32/32)	(35/29) (no details)	NR	PIM (1997)	DHI (20 mL ivgtt qd)	14d	TER	5
Chen et al. (2016)	RCT	80 (40/40)	NR	NR	NR	DHI (40 mL ivgtt qd)	NR	Hs-CRP	5
Huo et al. (2019)	RCT	80 (40/40)	(28/12): (26/14)	(62 ± 4): (61 ± 6)	TUDMI (2013)	DHI (40 mL ivgtt qd)	14d	IHF, CK-MB, hs-CRP	5
Zhang et al. (2010)	RCT	214 (110/104)	(142/72) (no details)	NR	NDCIHD (WHO)	DHI (40 mL ivgtt qd)	14d	IA	5
Zhang and Sun (2009)	Non-RCT	100	(63/37) (no details)	NR	DCAMI (1979 WHO)	DHI (30 mL ivgtt qd)	14d	TER	4
Qin (2015)	RCT	220 (110/110)	(60/50): (64/46)	(67.5 ± 1.9): (64.2 ± 3.1)	NR	DHI (30 mL ivgtt qd)	14d	TER	5
Chen (2015)	RCT	108 (54/54)	(60/48) (no details)	NR	NR	DHI (30 mL ivgtt qd)	14d	TER	6
Lei (2014)	RCT	110 (55/55)	(30/25): (32/23)	(68.8 ± 2.6): (66.8 ± 2.6)	NR	DHI (40 mL ivgtt qd)	14d	TER	5
Feng (2021)	RCT, Open-blinding	158 (79/78)	(40/39): (42/36)	(60.19 ± 1.38): (60.25 ± 1.2)	NR	DHI (ivgtt qd)	7d	LVEF, ADRs	5
Shi (2018)	RCT	80 (40/40)	(25/15): (27/13)	(68.27 ± 2.89): (67.12 ± 2.20)	NR	DHI (12 mL ivgtt qd)	14d	TER	5
Jiang et al. (2011)	Non-RCT	82 (42/40)	(31/11): (30/10)	(666.1 ± 11.6): (65.9 ± 10.8)	DCCHD (WHO)	DHI (30 mL ivgtt qd)	20d	TER	4

RCT, randomized controlled trials; T, treatment group; C, control group; M, male; F, female; NR, not report; GDMAMI (2001 CMA), Guidelines for the diagnosis and management of acute myocardial infarction (2001 Chinese Medical Association); DCAMI (1979 WHO), Diagnostic criteria for acute myocardial infarction (1979 World Health Organization); NDCIHD (WHO), Nomenclature and diagnostic criteria for ischemic heart disease (World Health Organization); DCCHD (WHO), Diagnostic criteria for coronary heart disease (World Health Organization); PIM (1997), Practical internal medicine; UNMIDT (2007 CMA), Unstable angina and non-ST-segment elevation myocardial infarction diagnosis and treatment guidelines (2007 Chinese medical association); DTSTEMI (2010), Diagnosis and treatment of acute ST-segment elevation myocardial infarction (2010); DTSTEMI (2015), Diagnosis and treatment of acute ST-segment elevation myocardial infarction (2015); CM (2003), Cardiovascular Medicine; CHD (2003), Coronary heart disease; CM (1980 NCPGIM), Coronary heart disease (1980 National Cardiovascular Professional Group of Internal Medicine); GTNSTE-ACS (2012), Guidelines for diagnosis and treatment of non-ST elevation acute coronary syndrome (2012); DCASTEMI, Diagnostic criteria of acute non-ST elevation myocardial infarction; TUDMI (2013), Third universal definition of myocardial infarction (2013); TER, Total effective rate; ADRs, Incidence of adverse reactions; LVEF, Left ventricular ejection fraction; IA, Incidence of arrhythmia; CK-MB, Creatine kinase isoenzyme; hs-CRP, Hypersensitive C-reactive protein; IHF, Incidence of heart rhythm failure; ICS, Incidence of cardiogenic shock; RBV, Rate of blood vessels; CFR, Case fatality rate.

### Quality evaluation of study quality

**FIGURE 2:**
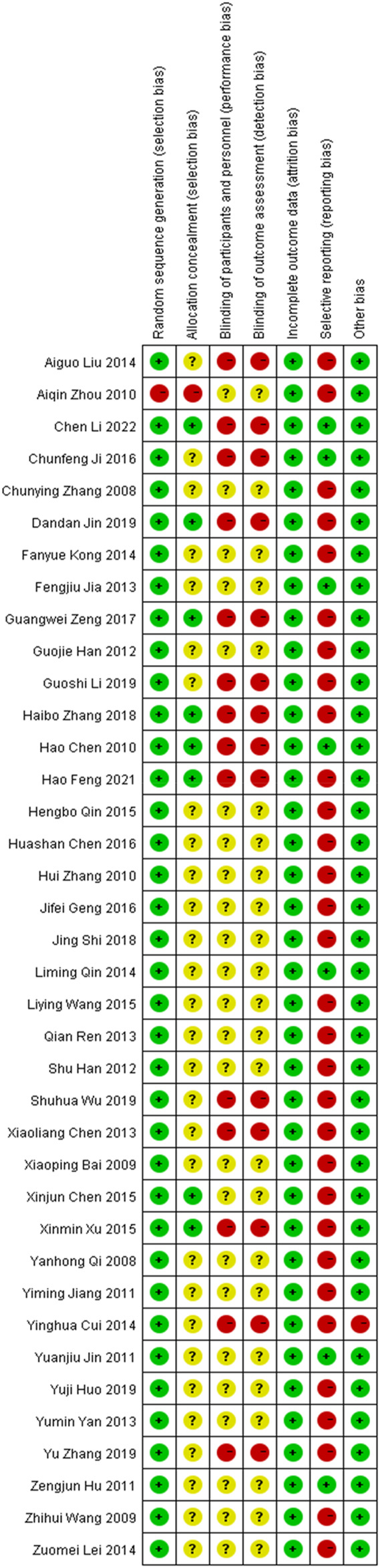
Schematic diagram of risk assessment of bias.

**FIGURE 3:**
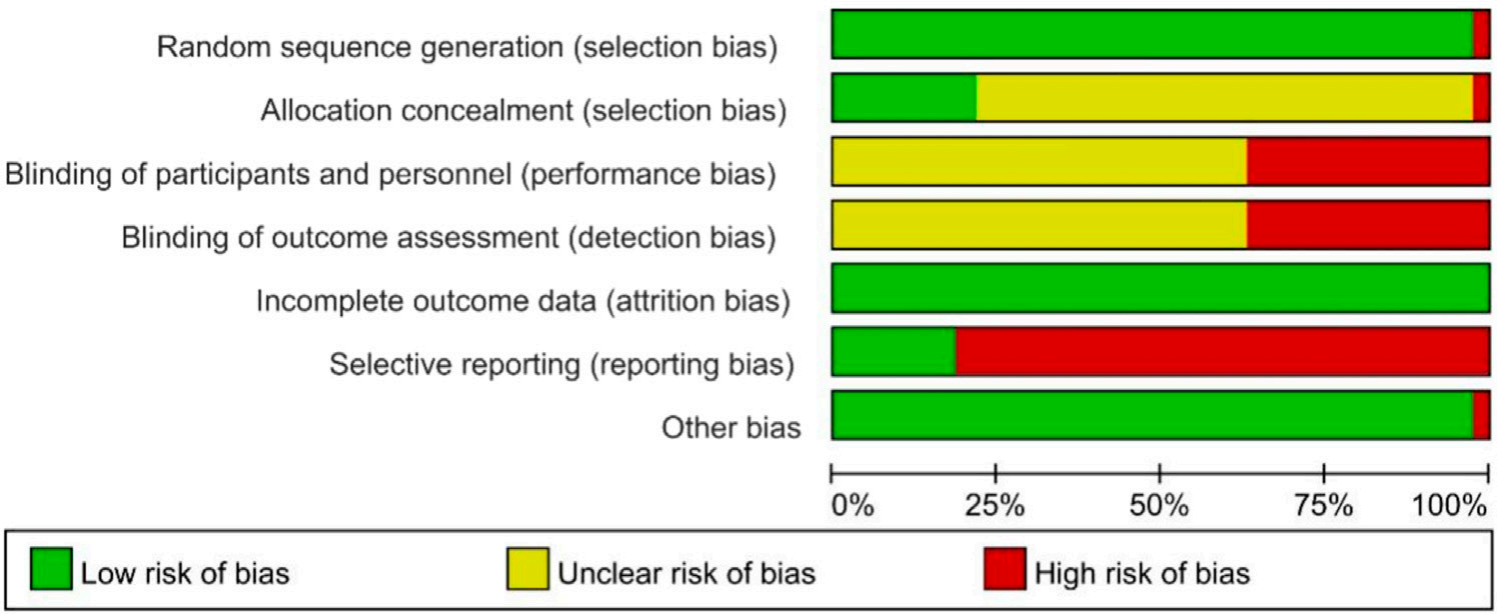
Proportion of risk assessment of bias.

A total of 38 studies (Qi et al., 2008; Bai et al., 2009; Wang et al., 2009; Zhang and Sun, 2009; Chen et al., 2010; Zhang et al., 2010; Zhou et al., 2010; Hu et al., 2011; Jiang et al., 2011; Jin et al., 2011; Han et al., 2012; Han and Wang, 2012; Chen and LIU, 2013; Jia et al., 2013; Ren and Jia, 2013; Yan, 2013; Cui and Wang, 2014; Kong and Zhao, 2014; Lei, 2014; Liu and Gu, 2014; Qin et al., 2014; Chen, 2015; Qin, 2015; Wang et al., 2015; Xu et al., 2015; Chen et al., 2016; Geng and Hu, 2016; Ji et al., 2016; Zeng et al., 2017; Shi, 2018; Zhang et al., 2018; Zhang et al., 2019; Huo et al., 2019; Jin et al., 2019; Li et al., 2019; Wu, 2019; Feng, 2021; Li et al., 2022) were included. All studies were RCT-s, with six studies using random number tables, one study using random coin tosses, one study using random sampling, and the remaining studies only referring to randomization. The schematic diagram of the risk assessment of bias included in the studies is shown in Figure 2, and the percentage of the risk assessment of bias is shown in Figure 3.

## Outcomes

### Total effective rate

**FIGURE 4:**
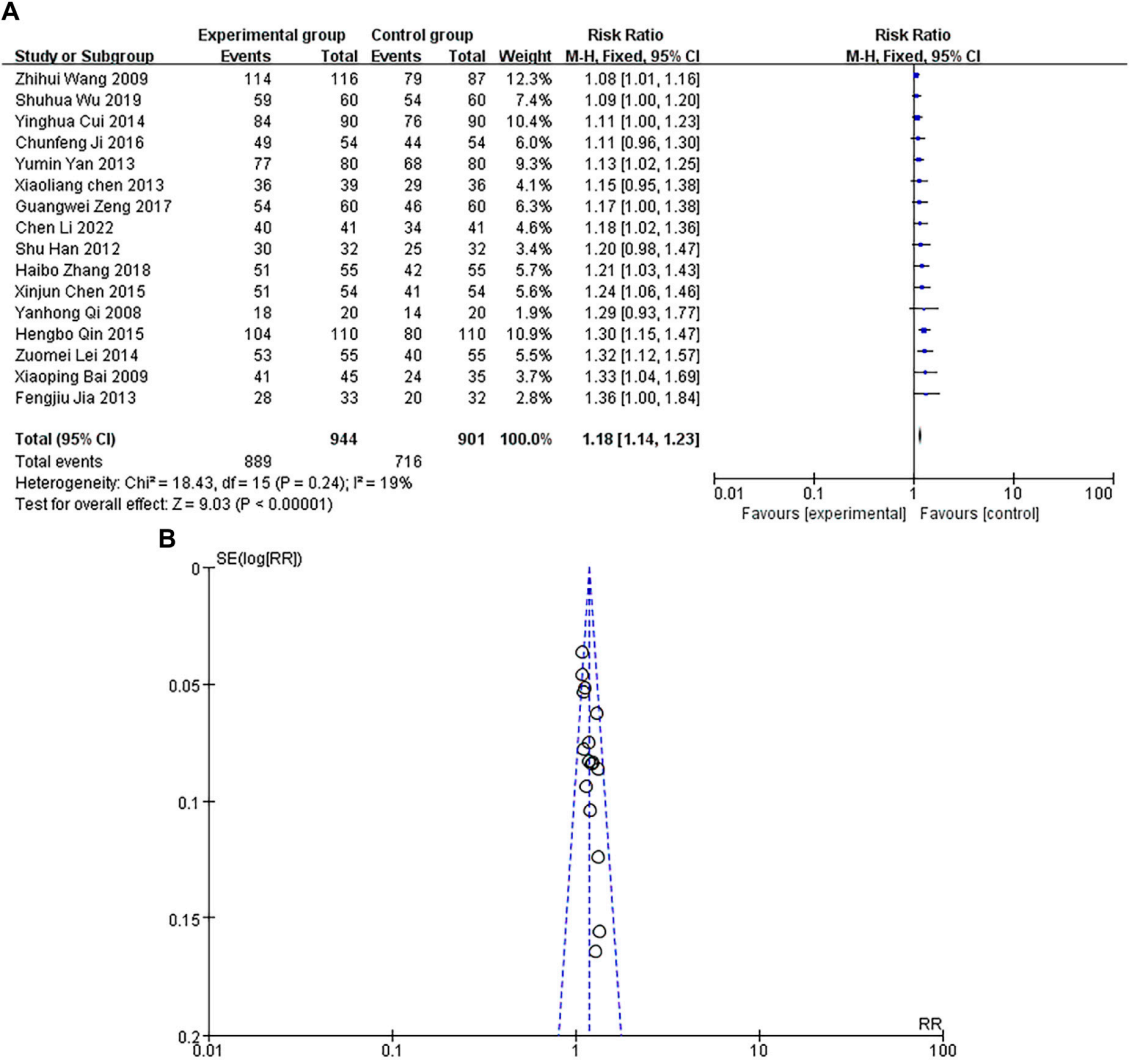
**(A)** Forest plot of Total effective rate. **(B)** Funnel plot of total effective rate.

A total of 16 studies were included, with a heterogeneity test of *p* = 0.24 > 0.1, I^2^ = 19% < 50%, indicating that there was no heterogeneity in the included studies. The results of fixed-effect pooled effect size (Li et al., 2020) showed that the RR and 95% CI were 1.18 [1.14–1.12], *p* < 0.05, and the difference in results was statistically significant, as shown in Figure 4A. The sample size of the study is large, and its corresponding points are mainly close to the narrow area in the middle and upper part of the funnel. There is no heterogeneity or other bias in the results, as shown in Figure 4B. The results showed that the total effective rate of Danhong injection in the experimental group was higher than that of conventional treatment in the control group.

### Incidence of arrhythmia

**FIGURE 5:**
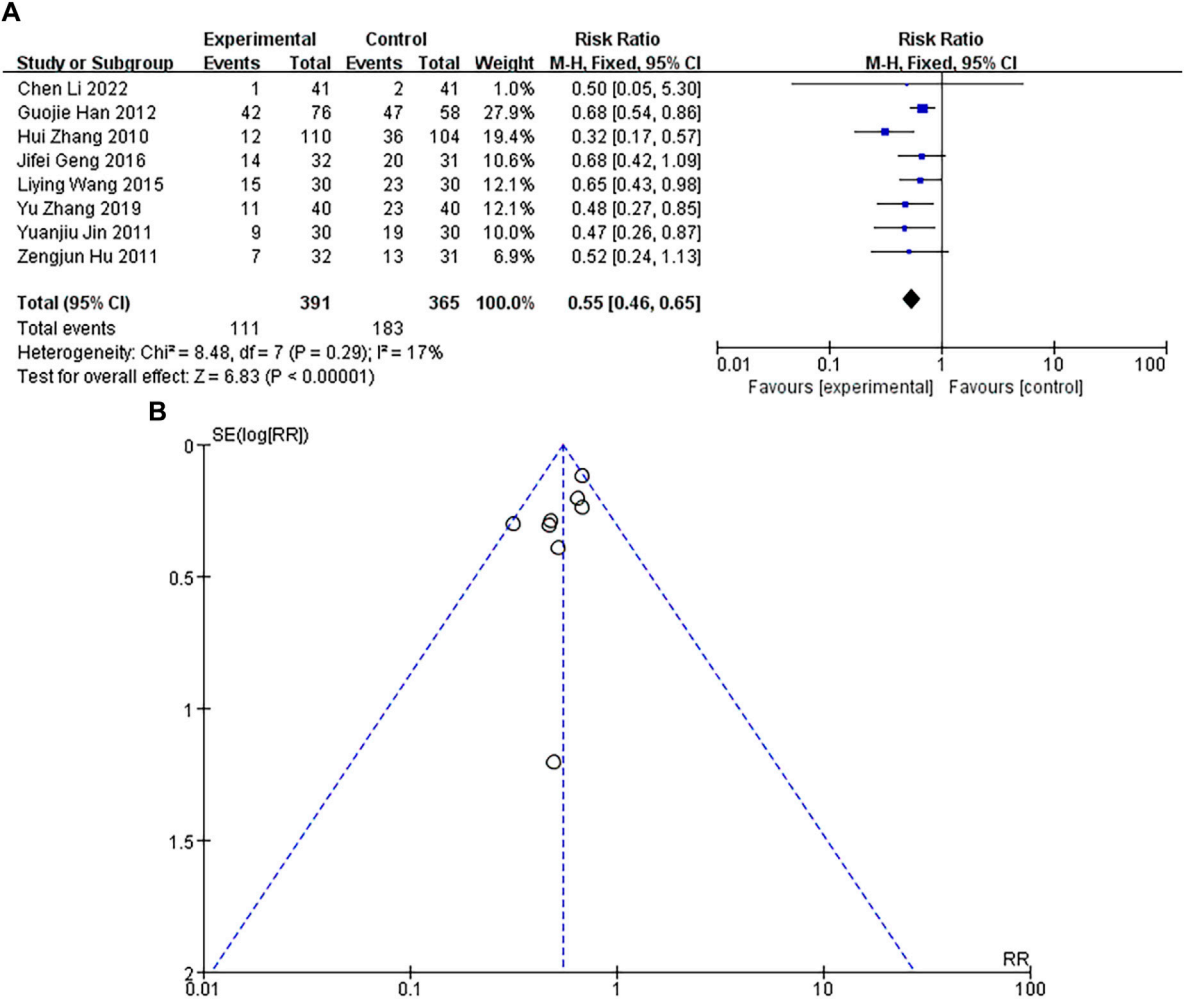
**(A)** Forest plot of arrhythmia incidence. **(B)** Funnel plot of arrhythmia incidence.

A total of 8 studies were included, with a heterogeneity test of *p* = 0.29 and I^2^ = 17%, indicating that there was no heterogeneous in the included studies were considered to be. The results of fixed-effect pooled effect size showed that the RR and 95% CI were 0.55 [0.46–0.65], *p* < 0.05, and the difference in results was significant and statistically significant, as shown in Figure 5A. The sample size of the study is large, and its corresponding points are mainly clustered in the narrow area of the upper funnel. There is no heterogeneity and other bias in the results, as shown in Figure 5B. The results showed that the incidence of arrhythmia in the experimental group was lower than that in the control group.

### Incidence of heart rhythm failure

**FIGURE 6:**
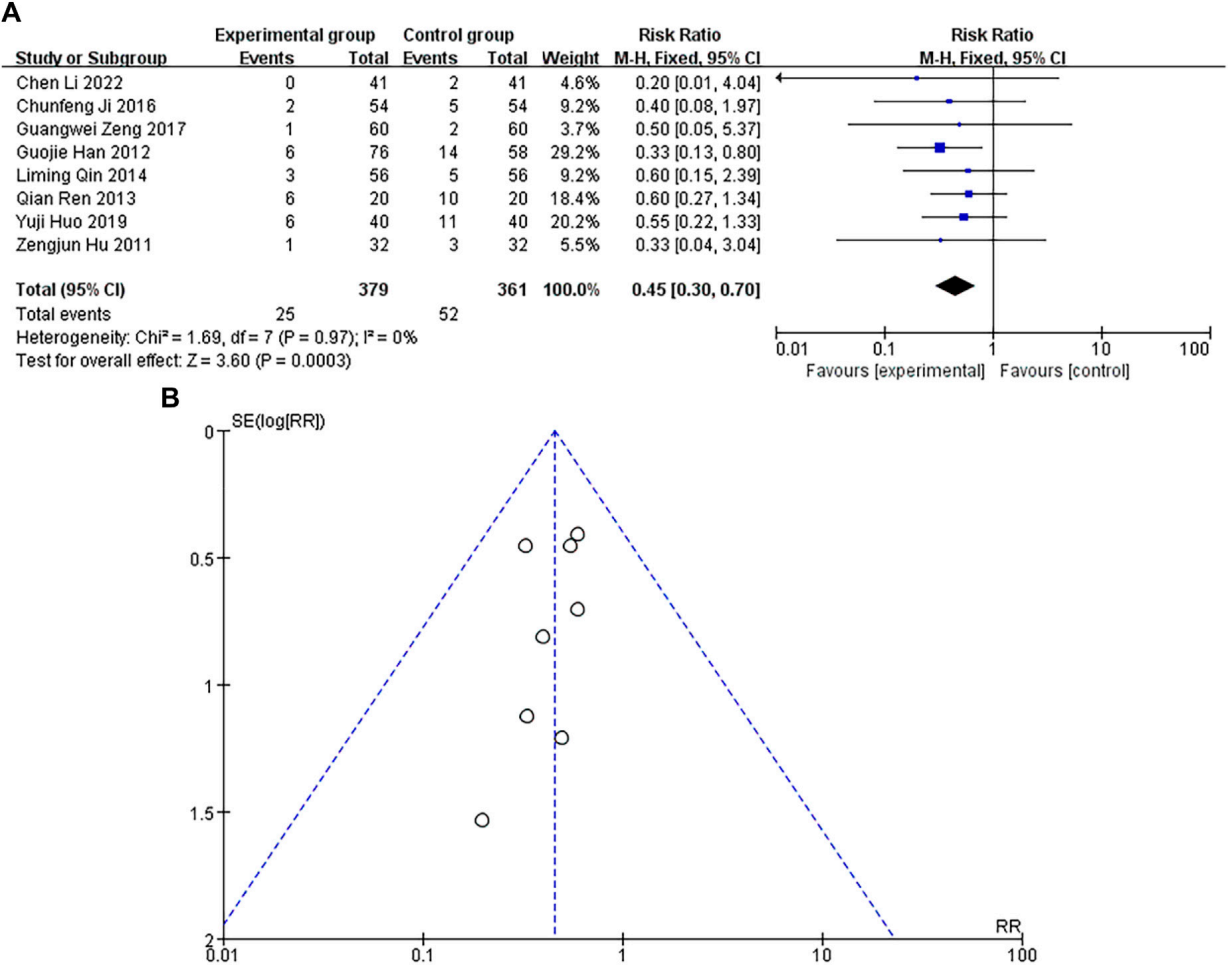
**(A)** Forest plot of heart rate failure incidence. **(B)** Funnel plot of heart rate failure incidence.

A total of 8 studies were included, with a heterogeneity test of *p* = 0.97 and I^2^ = 0%, indicating that there was no heterogeneity in the included studies. The results of fixed-effect pooled effect size showed that the RR and 95% CI were 0.45 [0.30–0.70], *p* < 0.05, and the difference in results was significant and statistically significant, as shown in Figure 6A. The sample size of the study is large, and its corresponding points are mainly close to the middle and upper regions of the funnel. There is no heterogeneity or other bias in the results, as shown in Figure 6B. The results showed that the incidence of heart rhythm failure in the experimental group was lower than that in the control group.

### Incidence of cardiogenic shock

**FIGURE 7:**
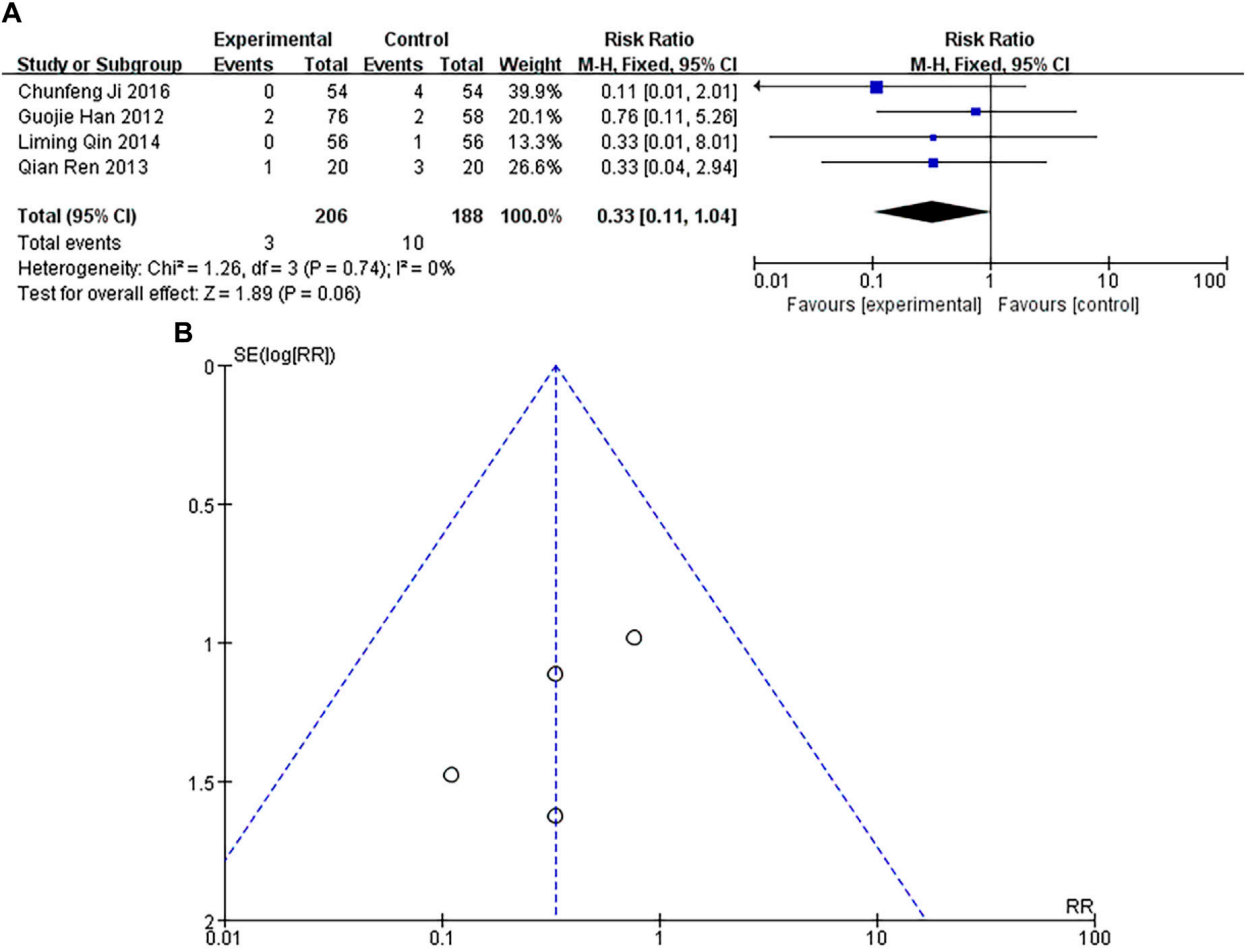
**(A)** Forest plot of cardiogenic shock. **(B)** Funnel plot of cardiogenic shock.

A total of 4 studies were included, with a heterogeneity test of *p* = 0.74 and I^2^ = 0%, indicating no heterogeneous in the included studies. The results of fixed-effect pooled effect size showed that the RR and 95% CI were 0.33 [0.11–1.04], *p* = 0.06 > 0.05, and there was no significant difference in the results, which was not statistically significant, as shown in Figure 7A. The number of studies was small and the sample size was small, and their corresponding points were mainly scattered in the middle and lower region of the funnel. There was no heterogeneity in the results, but there were other biases, as shown in Figure 7B.

### Left ventricular ejection fraction (LVEF)

**FIGURE 8:**
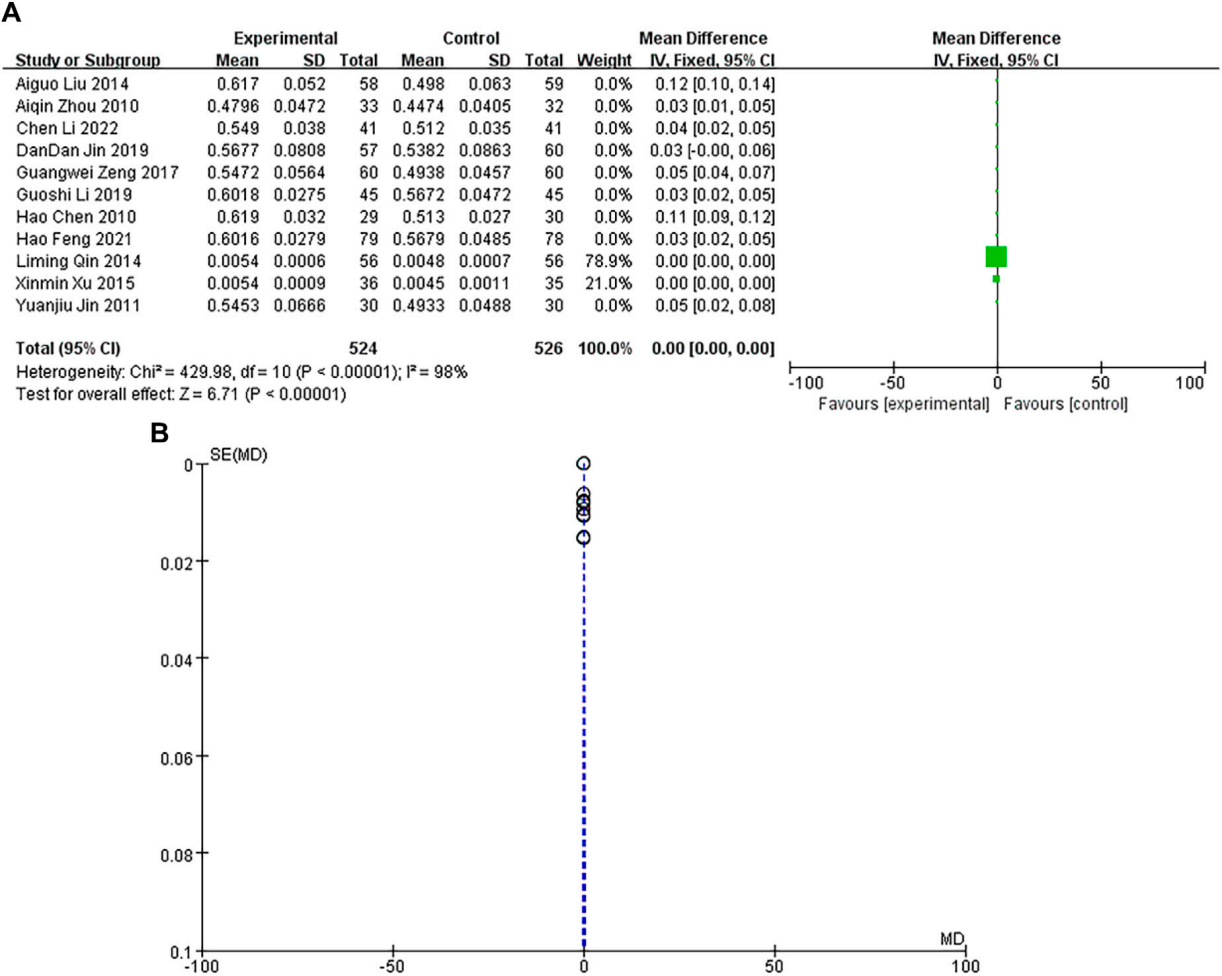
**(A)** LVEF forest diagram. **(B)** LVEF funnel diagram.

A total of 11 studies were included, with a heterogeneity test of *p* < 0.00001 and I^2^ = 98% > 50%, indicating that there was heterogeneity in the included studies. Using the random-effects model, the pooled results showed that the MD and 95% CIs were 0.00 [0.00–0.00], *p* < 0.00001, and there was no significant difference in the results, which was not statistically significant, as shown in Figure 8A. The sample size of the study is large, and its corresponding points are mainly close to the narrow area in the upper part of the funnel. There are heterogeneity and other biases in the results, as shown in Figure 8B. The results showed that there was no difference in LVEF between the experimental group and the control group.

### Creatine kinase isoenzyme (CK-MB)

**FIGURE 9:**
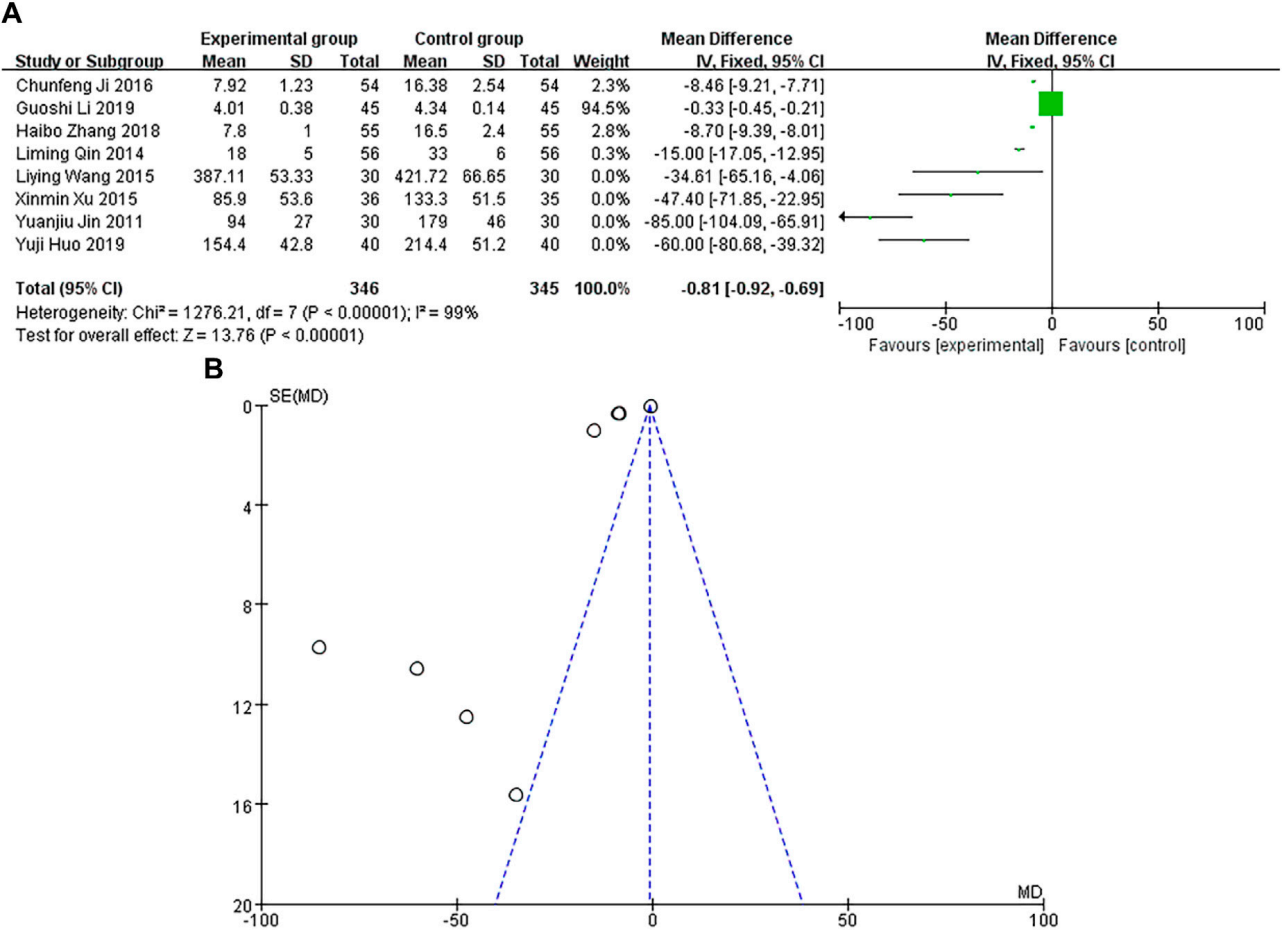
**(A)** CK-MB forest diagram. **(B)** CK-MB funnel diagram.

A total of 8 studies were included, with the heterogeneity test of *p* < 0.00001 and I^2^ = 99% > 50%, indicating that the included studies were heterogeneous. Using the random-effects model, the pooled results showed that MD and 95% CI were −0.81 [−0.92∼−0.69], *p* < 0.00001, and the difference in results was significant and statistically significant, as shown in Figure 9A. The sample size of the study is relatively scattered, and its corresponding points are distributed in the area outside the funnel plot. There are heterogeneity and other biases in the results, as shown in Figure 9B. The results showed that the CK-MB of the experimental group was lower than that of the control group.

### Hypersensitive C-reactive protein (hs-CRP)

**FIGURE 10:**
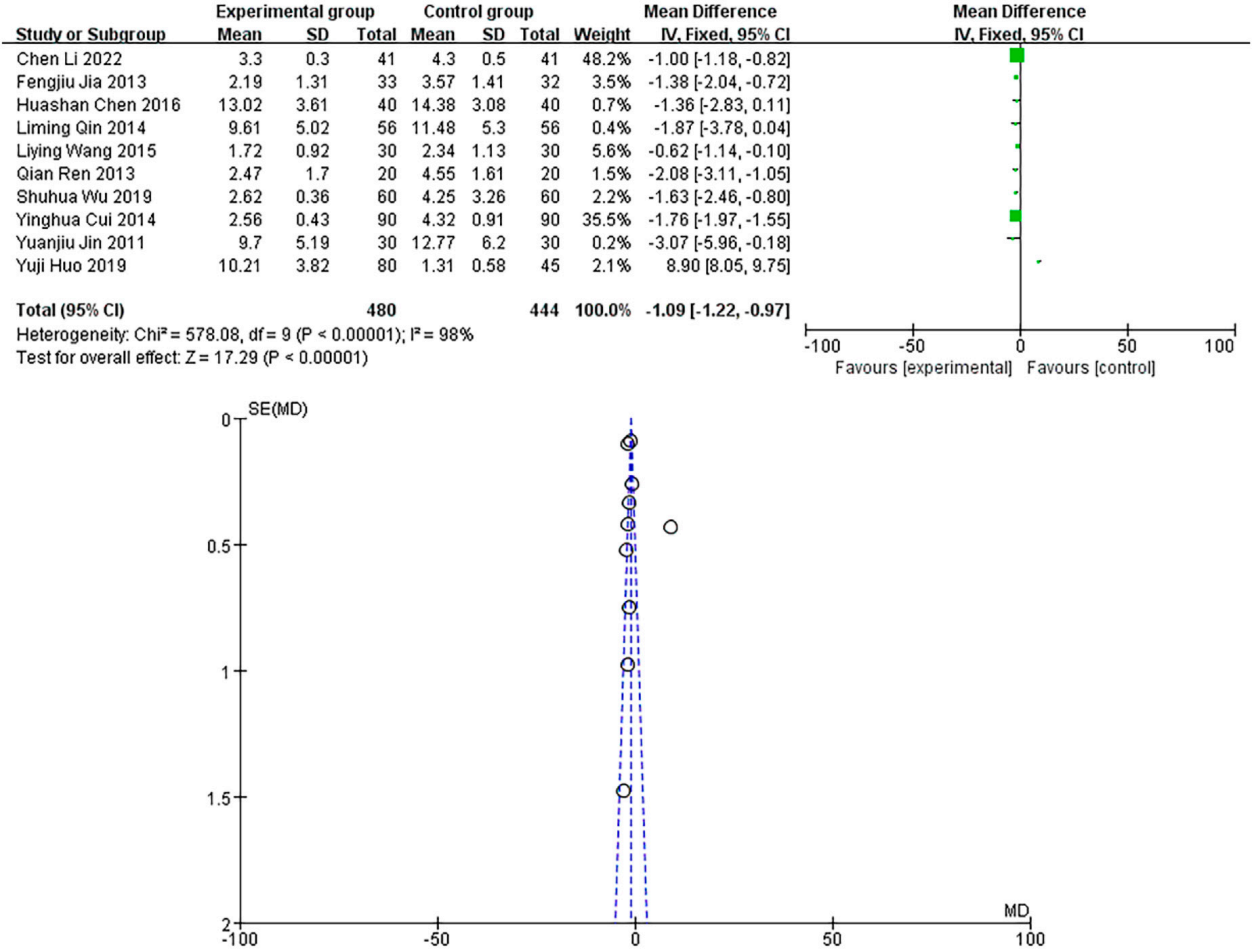
**(A)** hs-CRP forest diagram. **(B)** hs-CRP funnel diagram.

A total of 10 studies were included, with a heterogeneity test of *p* < 0.00001 and I^2^ = 98% > 50%, indicating that the included studies were heterogeneous. Using the random-effects model, the pooled results showed that the MD and 95% CIs were −1.09 [−1.22∼−0.97], *p* < 0.00001, and the difference in results was significant and statistically significant, as shown in Figure 10A. The sample size of the study is relatively scattered, and the corresponding points are mainly close to the narrow area in the upper part of the funnel plot. There are heterogeneity and other biases in the results, as shown in Figure 10B. The results showed that the hs-CRP of the experimental group was lower than that of the control group.

### Incidence of adverse reactions

**FIGURE 11:**
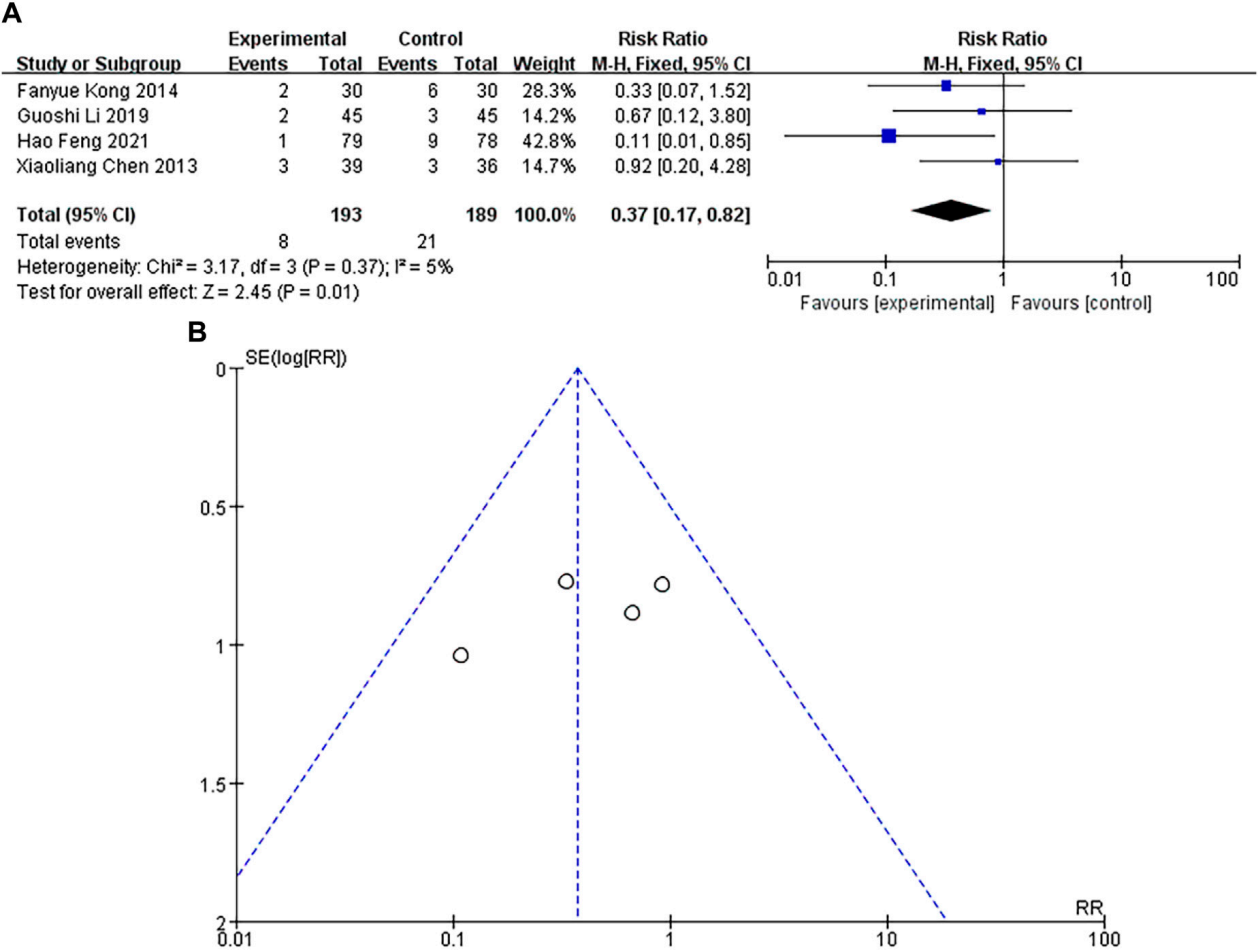
**(A)** Forest plot of incidence of adverse reactions. **(B)** Funnel plot of incidence of adverse reactions.

A total of 10 studies were included, with a heterogeneity test of *p* = 0.37 and I^2^ = 5%, and it was considered that there was no heterogeneity in the included studies. The results of fixed-effect pooled effect size showed that the RR and 95% CI were 0.37 [0.17–0.82], *p* < 0.05, and the difference in results was significant and statistically significant, as shown in Figure 11A. The number of studies is small, the sample size is large, and the corresponding points are mainly close to the middle and upper regions of the funnel. There is no heterogeneity and other bias in the results, as shown in Figure 11B. The results showed that the incidence of adverse reactions in the experimental group was lower than that in the control group.

## Discussion

Routine meta-analysis was performed on 38 RCTs according to the PRISMA guidelines. Revman software was used to perform statistical analysis of the effect size pooling. Meta-analysis results showed that DHI can not only improve the total effective rate of MI, but also reduce the incidence of arrhythmia, heart rate failure, CK-MB, hs-CRP and adverse reactions. It is suggested that DHI can effectively improve cardiac function, promote blood regurgitation and reduce the incidence of adverse reactions.

The overall effect of the test using the fixed-effect model showed that compared with the control group, the total effective rate was Z = 9.03 (*p* < 0.00001), confirming the effectiveness and safety of DHI in treating MI. The incidence of arrhythmia was Z = 6.83 (*p* < 0.00001), confirming the antioxidant and cardioprotective effects of Danshen on CVD (Bu et al., 2019). Pharmacological studies have shown that Safflower extract in DHI can reduce the incidence of arrhythmias by altering calcium overload of myocardial cells (Yu et al., 2022). The incidence of heart failure was Z = 3.60 (*p* = 0.0003), verifying that DHI can reduce cardiac load and improve myocardial tissue remodeling (Zheng et al., 2024). The incidence of adverse reactions was Z = 2.45 (*p* = 0.01), and the possible adverse reactions were related to the speed of DHI intravenous infusion and the patient’s allergy history (Gu X.L., 2022). The overall effect of random effect model test showed that CK-MB was Z = 13.76 (*p* < 0.00001), indicating that DHI has a certain improvement effect on myocardial injury. hs-CRP was Z = 17.29 (*p* < 0.00001), confirming that DHI had a certain inhibitory effect on inflammatory response. Our studies have proved that DHI has a significant therapeutic effect in the treatment of MI, which can effectively improve cardiac function, reduce the incidence of adverse reactions, and improve the overall living standard of patients.

MI, commonly known as a heart attack, is the main cause of death in patients with CVD (Chen et al., 2022), with the most common cause being a decrease or cessation of blood flow to the heart. At present, China is one of the countries with the heaviest burden of CVD in the world (Zhao, 2019), and the turning point of disease burden reduction has not yet appeared (Ma et al., 2023). Over the past 40 years, our understanding of the pathogenesis of MI has continued to evolve, allowing for new treatment strategies to dramatically improve survival rates (Saleh and Ambrose, 2018). With the frequent and successful application of traditional Chinese medicine (TCM) in the prevention of CVD, the efficacy of TCM in the treatment of CVD has attracted more and more attention (Liu et al., 2011). For example, in recent years, DHI has been a commonly used method for treating MI, and a large number of clinical trials have not only revealed the positive therapeutic effect of DHI on MI patients (Cui et al., 2021), but also reflected its multiple mechanisms of action on CVD. DHI can reduce the production of serum C-reactive protein (CRP) by inhibiting the activity of NF-κB in the vessel wall, thereby alleviating local inflammatory response (Fu et al., 2009). At the same time, DHI also has varying degrees of antihypertensive effect (Yang et al., 2024). In addition, DHI can also protect vascular endothelium, promote angiogenesis, inhibit thrombin activity, and promote thrombolysis (Li et al., 2018). Depending on the etiology and condition, MI can be divided into 5 types, among which type 2 is mainly caused by an increase in oxygen demand (eg, hypertension) or a decrease in oxygen supply (eg, coronary spasm or embolism, arrhythmias, hypotension) (Thygesen et al., 2018). Research has found that DHI is more effective in treating type 2 MI, and there is no significant difference in the treatment of recurrent MI (Hu et al., 2019). The potent and widely used role of DHI has been confirmed in pharmaceutical research. For example, DHI has a positive effect on inhibiting platelet aggregation, improving hemodynamic status, and protecting endothelial function in the treatment of MI patients (Liu et al., 2015). The studies included in this meta-analysis did not mention the side effects of DHI, but DHI may have some side effects, such as skin itching, flushing, rash, swelling at the injection site, nausea, and vomiting (Yu et al., 2018).

In clinical practice, TCM injections has been widely used in the treatment of MI in China and other Asian countries (Wang et al., 2018). As a composite endpoint of reinfarction and stroke, acute heart failure, and re-hospitalization for CVD, research on TCM injections has entered the stage of molecular biology (Gong et al., 2023). For the treatment of MI, TCM has the following principles of “treating the surface when urgent, and treating the root when slow”. The former is a life-saving measure, while the latter is the long-term maintenance of the stent to facilitate cardiac recovery. MI belongs to the category of heart pain, true heart pain, and chest pain in traditional Chinese medicine. In DHI, Danshen has the function of nourishing blood promote blood circulation and remove blood stasis, and it is the main medicine; Safflower has the effect of promoting blood circulation, removing blood stasis, and relieving pain, and is used as an auxiliary medicine. Therefore, DHI has the effects of promoting blood circulation and removing blood stasis, clearing meridians and soothing collaterals, and is used for chest pain caused by blood stasis and obstruction (Feng et al., 2019). DHI have been reported as a potential effective method for treating MI, but there are still many difficulties in its use, such as a lack of scientific evidence that has been rigorously evaluated by clinical trials, complex components, and unclear pharmacological mechanisms, which often hinder the promotion and use of TCM products in Western countries (Wang et al., 2018). At present, there are few systematic analysis studies on DHI in the treatment of MI, so it is necessary to systematically evaluate the efficacy and safety of DHI in the treatment of MI.

## Limitations

There are some limitations in this study. 1) Of the 38 included studies, only 8 provided detailed descriptions of the implementation of randomization. 2) Only 14 of the 38 studies explicitly mentioned the use of blinding. 3) There were differences in the experimental designs of the included studies. 4) Due to the low quality of the included studies, the widespread popularity of TCM and the preference of Chinese patients for TCM, it is difficult to distinguish whether the effect of DHI is confused by the effect of other TCM drugs, which will bring some bias to the meta-analysis. 5) Due to the small number of studies included in the statistical analysis and the small follow-up effect size, the upper and lower limits of 95% CI for the binary variable cardiogenic shock included 1, and the upper and lower limits of 95% CI for the continuous variable LVEF included 0, and the results showed no significant difference or statistical significance, so the conclusion of this study was not perfect.

## Conclusion

In summary, this study conducted a meta-analysis of the treatment of MI with Danhong injection, and concluded that Danhong injection had significant efficacy in the treatment of MI. Compared with conventional treatment, Danhong injection has a benefit in patients with MI in terms of total response rate, arrhythmias, heart failure, CK-MB and hs-CRP incidence, effectively improving cardiac function, while reducing the incidence of adverse effects and improving overall quality of life.

## Data Availability

The original contributions presented in the study are included in the article, further inquiries can be directed to the corresponding author.
